# Association of minimal residual disease with clinical outcomes in Philadelphia chromosome positive acute lymphoblastic leukemia in the tyrosine kinase inhibitor era: A systemic literature review and meta-analysis

**DOI:** 10.1371/journal.pone.0256801

**Published:** 2021-08-26

**Authors:** Wanhua Zhang, Erguai Jang

**Affiliations:** 1 Department of Hematology, West China Hospital, Sichuan University, Chengdu, Sichuan Province, China; 2 West China School of Medicine, Sichuan University, Chengdu, Sichuan Province, China; University of Texas at Austin, UNITED STATES

## Abstract

Minimal residual disease (MRD) appeared to be a potent prognostic indicator in patients with Philadelphia chromosome positive acute lymphoblastic leukemia (Ph+ ALL), with potential value in informing individualized treatment decisions. Hence, we performed herein a systemic literature review and meta-analysis to comprehensively address the prognostic value of MRD in Ph+ ALL. Systematic literature review was conducted in PubMed, Embase, and Cochrane databases with the data access date up to September 23, 2020. Pooled hazard ratios (HRs) with 95% confidence intervals (CIs) were calculated with fixed-effects or random-effects models. Furthermore, subgroup analyses were performed to assess the robustness of the associations. 27 studies with a total number of 3289 patients were eligible for this meta-analysis. Combined HRs suggested that MRD positivity was associated with inferior event-free survival (EFS) (HR = 2.00, 95% CI 1.77–2.26) and overall survival (OS) (HR = 2.34, 95% CI 1.86–2.95). The associations remained statistically significant in subgroup analyses including age group, MRD timing, disease status at MRD, MRD cutoff level, et al. Our findings suggested MRD as a potent clinical tool for assessing the prognosis of Ph+ ALL. Further studies using MRD-based risk stratification might help optimize individualized treatment strategies for Ph+ ALL patients.

## Introduction

Philadelphia (Ph) chromosome is the der(22) product of the reciprocal translocation between 9q34 and 22q11.2, which generates the BCR-ABL1 fusion gene [1]. It can be detected in 25–40% of adult patients with acute lymphoblastic leukemia (ALL) [2, 3]. Historically, Philadelphia chromosome was associated with a dismal prognosis, with long-term survival rates of less than 20%, and the only curative possibility was based on intensive chemotherapy followed by allogeneic hematopoietic stem cell transplantation (allo-HSCT) [4–6]. The addition of tyrosine kinase inhibitors (TKIs) has remarkably improved the treatment response and long-term survival of Ph+ ALL patients [7–12]. However, a substantial proportion of patients still die as a result of disease progression. Thus, prediction and intervention before hematological relapse are important in reducing the incidence of relapse and improving clinical outcomes.

Minimal residual disease (MRD) refers to the persistence of residual leukemic cells that cannot be detected by conventional morphological methods [13]. It is a powerful prognostic factor in ALL and is used for patient stratification and treatment decisions, but its precise role in Ph+ ALL is less clear. In the TKI era, a plenty of studies have investigated the prognostic significance of MRD in Ph+ ALL patients. However, timing of MRD analysis, cutoff levels and treatment strategies varied across different studies, and consensus is still yet to be reached. Furthermore, Ph+ ALL differs from Ph- ALL not just in prognosis, but also in treatment regimens and method of MRD assessment. Hence, we performed herein a systemic literature review and meta-analysis to comprehensively explore the impact of MRD status in Ph+ ALL patients.

## Methods

### Literature search and study selection

This meta-analysis was performed and reported according to the PRISMA statement [14]. A PRISMA checklist (S1 Checklist) was used to ensure standardized reporting. The review protocol has been registered in the PROSPERO international prospective register of systematic reviews (registration number: CRD42021233397).

PubMed, Embase and Cochrane databases were searched for studies with the data access date up to September 23, 2020 with free-style words and Medical Subjects Headings (MeSH): (("Philadelphia Chromosome"[Mesh] OR BCR-ABL1) AND "Precursor Cell Lymphoblastic Leukemia-Lymphoma"[Mesh]) OR Ph positive acute lymphoblastic leukemia OR BCR-ABL1 positive acute lymphoblastic leukemia. The search strategy for Pubmed was provided in S1 File. References of included studies in this meta-analysis and relevant reviews were also screened for potentially eligible studies.

Two reviewers independently evaluated the eligibility of studies according to the predefined inclusion criteria: (1) randomized controlled trials (RCT) or cohort studies that investigated the association between MRD and prognosis in Ph+ ALL patients treated with regimens containing TKIs; (2) the study outcomes were time-dependent endpoints, such as disease-free survival (DFS)/relapse-free survival (RFS)/leukemia-free survival (LFS)/progression-free survival (PFS)/event-free survival (EFS) and overall survival (OS); (3) hazard ratios (HRs) with 95% confidence intervals (CIs) were reported or could be calculated from sufficient data presented. Studies were excluded if they: (1) included patients not treated with TKIs; (2) included less than 50 patients; (3) without sufficient data to calculate HRs and 95% CIs. For overlapped cohorts, only the latest and most intact report was included.

### Data extraction and quality assessment

Two reviewers independently extracted data from eligible studies using a standardized data collection sheet including the following items: first author, year of publication, study region, recruitment time, study design, treatment protocol, TKI used, transplant status, sample size, age range, ethnicity, sex distribution, follow-up time, MRD sample, MRD timing, disease status at MRD, pre-MRD treatment, post-MRD treatment, MRD test location, MRD detection method, MRD cutoff level, number of cases with positive MRD, outcomes, statistical methods, and HRs with 95% CIs.

The Quality in Prognosis Studies (QUIPS) tool [15] was used to assess the risk of bias of included studies in six domains: study participation, study attrition, prognostic factor measurement, outcome measurement, study confounding, statistical analysis and reporting. The risk of bias for each domain was indicated as low, moderate or high according to the rating criteria. The overall risk of bias for individual studies was marked as high if one or more domains were rated as high risk of bias, or moderate if one or more domains were rated as moderate risk of bias, or low if all domains were rated as low risk of bias.

Two reviewers independently performed the study screening, data extraction and quality assessment procedure. Any disagreements were resolved by consulting with a third reviewer.

### Statistical analyses

The primary endpoints were EFS and OS. DFS, RFS, LFS and PFS were interpreted as synonymous with EFS. HRs and 95% CIs for EFS and OS were pooled to assess the prognostic value of MRD. If both univariate and multivariate analyses results were presented, we used the latter. If HRs with 95% CIs were not reported, we estimated the data from Kaplan-Meier survival curves using the methods described by Tierney et al [16].

The heterogeneity between included studies was evaluated using chi-square based Q-test and *I*^*2*^ test. Random-effects model was applied to pool the HRs and 95% CIs if significant heterogeneity existed (P < 0.10 or *I*^*2*^ > 50%). Otherwise, fixed-effects model was used. Subgroup analyses were further performed to investigate the source of heterogeneity and to assess the potential effect modification of factors including study design, age group, study region, ethnicity, type of TKI used, transplant status, MRD timing, disease status at MRD, MRD cutoff level, and statistical method.

Publication bias was evaluated using Begg’s funnel plot and Egger’s test, with significance defined as P < 0.05. A trim-and-fill analysis was performed if publication bias was suggested. Sensitivity analyses were performed to evaluate single study’s influence on pooled HRs by sequentially omitting one study at a time. All of the analyses were performed with Stata version 16.0 software (Stata Corporation, College Station, TX, USA).

## Results

### Study selection and characteristics

**Fig 1:**
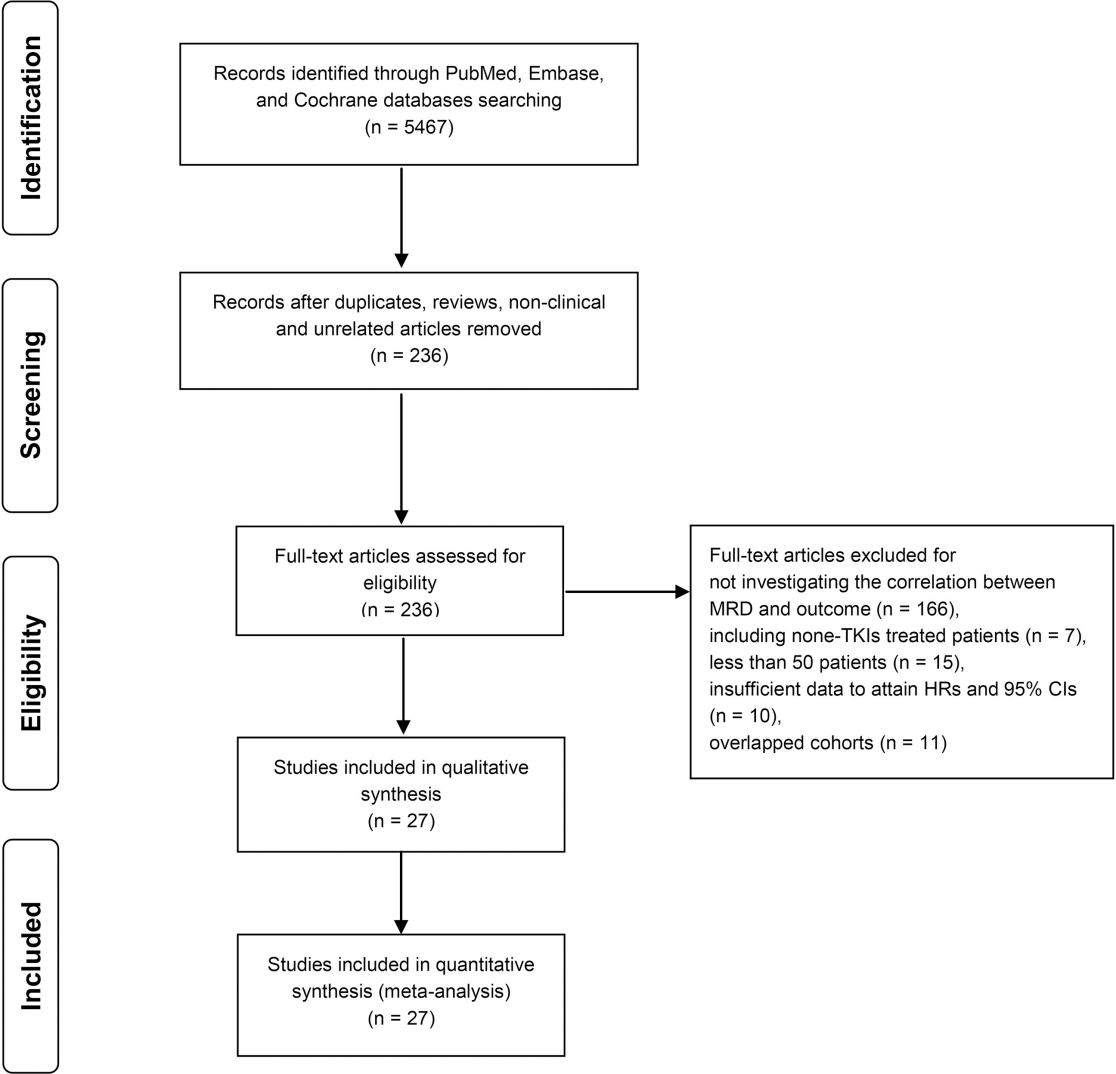
PRISMA flow diagram for study review and inclusion.

Initial literature searches in PubMed, Embase and Cochrane databases identified 5467 records published up to September 23, 2020. After screening the title and abstract of each record, 236 records were included in the full text screen. 209 records were discarded for the following reasons: not investigating the correlation between MRD and outcomes (n = 166), including non-TKIs treated patients (n = 7), less than 50 patients (n = 15), insufficient data to attain HRs and 95% CIs (n = 10), overlapped cohorts (n = 11). Finally, 27 records were included in the qualitative analysis [7, 9, 11, 17–40]. The study selection process is summarized as a flow chart in Fig 1.

The characteristics of included studies are summarized in S1 Table. The raw data is supplied in S2 File. Most studies were published from 2012 onwards, except for an RCT in Germany and two prospective cohort studies in Japan and Korea, spanning a time period from 2000 to 2019. Studies were mainly conducted in East Asia or Europe. The sample size ranged from 51 to 441, with a total number of 3289 patients. The age of patients was above 14 years in most studies. Only two studies included patients solely in the second complete remission (CR2) or later, and 22 studies were in patients in the first complete remission (CR1). Treatment protocols were typically multi-agent chemotherapy plus TKIs, followed by allo-HSCT, in most studies. Two studies applied CD19-targeted chimeric antigen receptor T cell (CAR-T) therapy for relapse/refractory patients. In ten studies patients all received HSCT, and five studies enrolled exclusively non-transplant patients, and the rest studies comprised both transplant and non-transplant individuals.

Almost all studies used BCR-ABL1 transcript level as a marker of MRD and used real-time quantitative polymerase chain reaction (RT-qPCR) to quantify the BCR-ABL1 transcript level, mostly based on bone marrow samples. The cutoff levels were set at 10^−5^, 10^−4^ or 10^−3^, or 4-log, 3-log or 1-log reduction from baseline. The measurements of MRD were generally taken within three months from induction treatment (n = 12) or before HSCT (n = 8).

According to the QUIPS tool, 15 studies were ranked as low risk of bias, and the rest were moderate risk of bias. The details are listed in S2 Table.

### Association between MRD status and EFS

**Fig 2:**
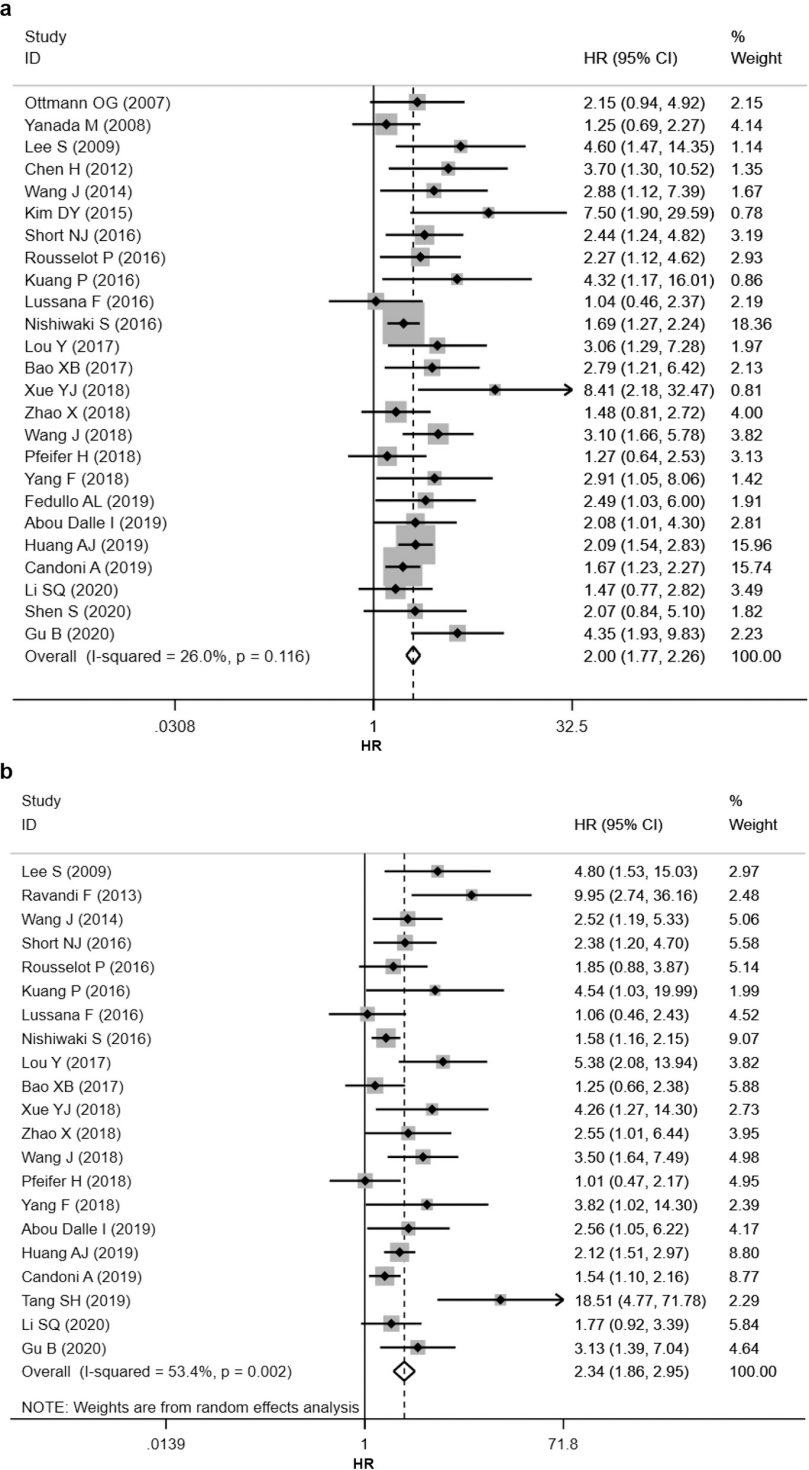
Forest plots for the impact of MRD status on the clinical outcomes of Ph+ ALL patients. (a) Forest plot for event-free survival (EFS). (b) Forest plot for overall survival (OS).

Fig 2A shows the meta-analysis result for EFS including 25 studies. Overall, MRD positivity was associated with worse EFS (HR = 2.00, 95% CI 1.77–2.26). The effect was consistent across all studies, with the confidence intervals crossing the null value in seven studies. No significant heterogeneity existed between the studies (*I*^*2*^ = 26.0%) and fixed-effects model was employed to pool the HRs.

In subgroup analysis, positive MRD was associated with inferior EFS in almost all subgroups (median age of 0–14, 15–39 or above 40 years; first- or second-generation TKIs treated patients; transplant or non-transplant cohort; MRD tested before or after three months from induction, before or after HSCT; different cutoff levels of 10^−5^, 10^−4^ or 10^−3^, or 4-log, 3-log or 1-log reduction from baseline; et al.), with non-significant confidence intervals in few subgroups (S3 Table). The prognostic value of MRD appeared to be stronger in the median age of 0–14 years (HR = 3.80, 95% CI 0.97–14.82) than in the 15–39 years (HR = 2.35, 95% CI 1.93–2.86) or ≥ 40 years subgroup (HR = 1.77, 95% CI 1.51–2.08). But it should be noted that the 0–14 years subgroup was only based on two studies and the confidence interval was very wide. The subgroup that received second-generation TKIs had a hazard ratio of 3.55 (95% CI 1.14–11.03), bigger than that of the first-generation TKI subgroup (HR = 2.03, 95% CI 1.65–2.50). Again it should be noted that the result of the second-generation TKIs subgroup was based on two studies, and their confidence intervals overlapped. The prognostic value of MRD also seemed to be stronger in ≥ CR2 patient cohorts (HR = 4.35, 95% CI 1.93–9.83), but this was only based on one study.

### Association between MRD status and OS

21 studies were included in the OS analysis. The overall result suggested inferior OS for patients with positive MRD (HR = 2.34, 95% CI 1.86–2.95) (Fig 2B), and the results were consistent across all studies. Moderate heterogeneity exited among the studies (*I*^*2*^ = 53.4%) and random-effects model was applied.

In subgroup analysis, MRD positivity remained a negative marker for OS. As for EFS, the prognostic value of MRD seemed to be stronger in the median age of 0–14 years subgroup (HR = 4.26, 95% CI 1.27–14.30) than in the 15–39 years (HR = 2.28, 95% CI 1.84–2.83) or ≥ 40 years subgroup (HR = 1.94, 95% CI 1.40–2.68). Similar to EFS, the 0–14 years subgroup was only based on one study and the confidence interval was very wide. The inferior effect was less notable in European patients (HR = 1.44, 95% CI 1.10–1.89) than in East Asian (HR = 2.68, 95% CI 2.00–3.59) or USA patients (HR = 3.01, 95% CI 1.83–4.96). In non-transplant cohorts, the prognostic value of MRD seemed to be greater (HR = 3.41, 95% CI 1.95–5.95) than in transplant cohorts (HR = 1.58, 95% CI 1.32–1.90).

### Publication bias and sensitivity analyses

**Fig 3:**
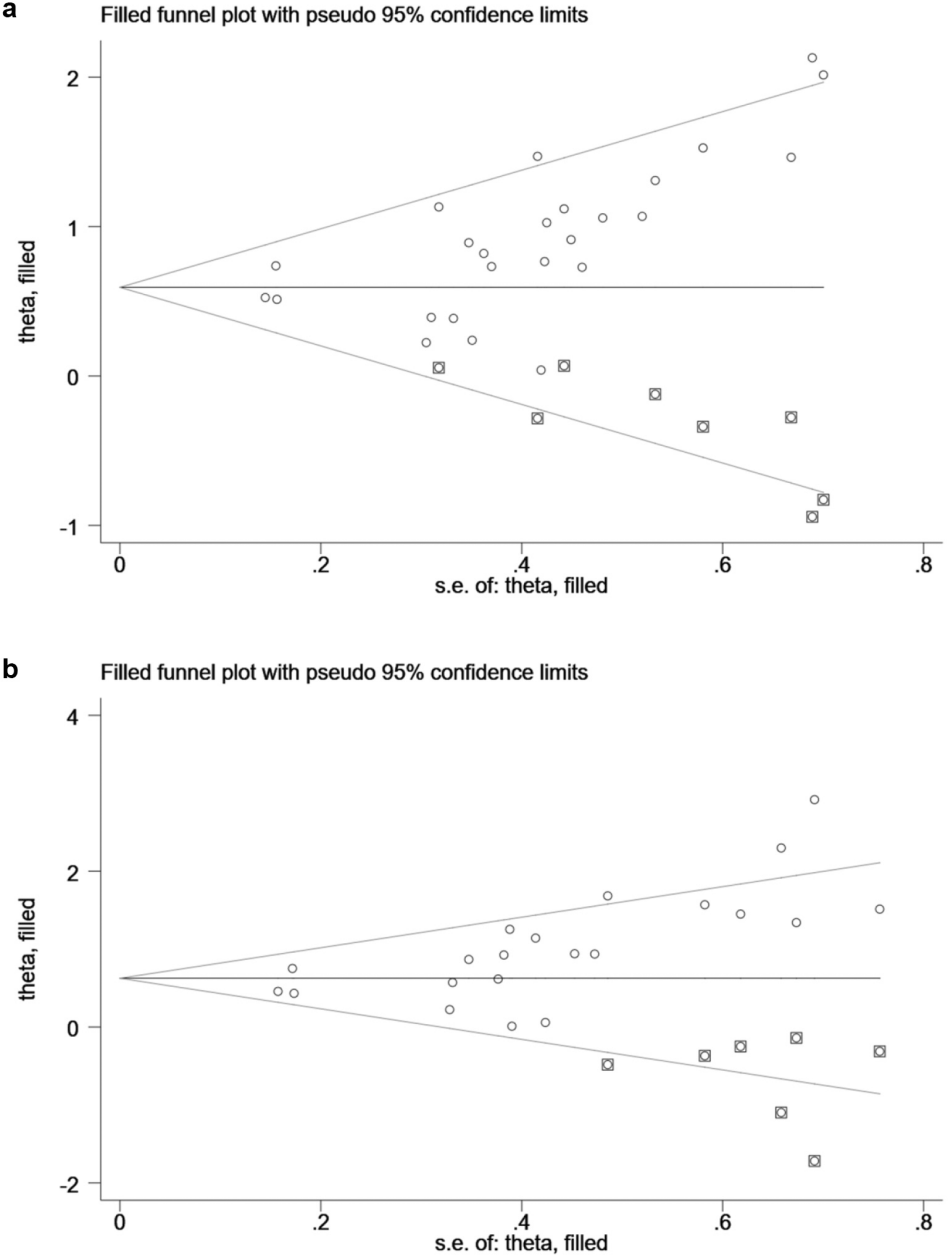
Begg’s funnel plot with trim-and-fill analysis for publication bias. (a) event-free survival (EFS) and (b) overall survival (OS). The circles in squares indicated the hypothesized studies, which were also incorporated to generate symmetrical funnel plots.

**Fig 4:**
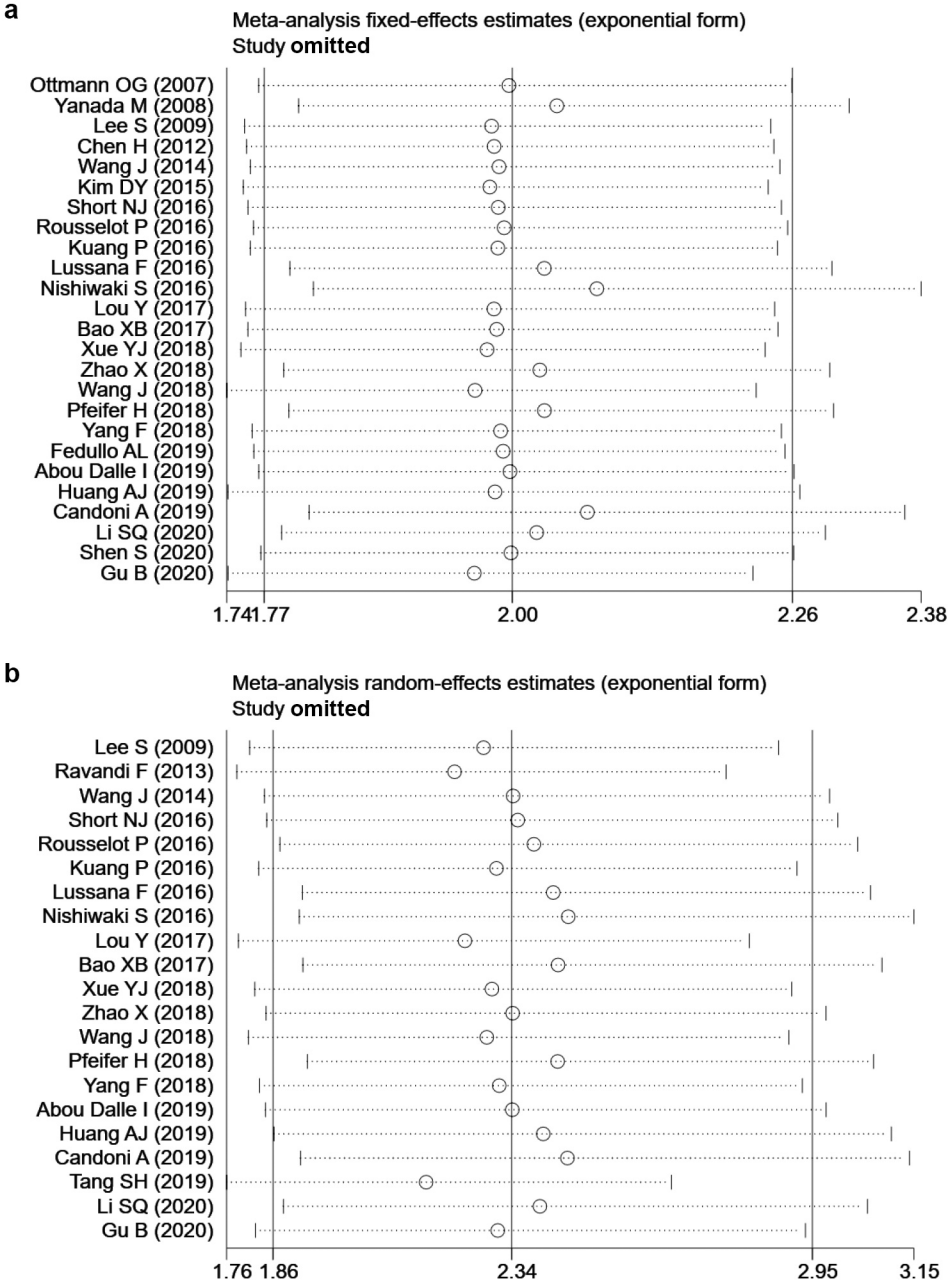
Sensitivity analysis for (a) event-free survival (EFS) and (b) overall survival (OS).

Begg’s and Egger’s test suggested potential risks of publication bias for EFS and OS. By trim-and-fill analyses, eight and seven hypothesized studies were imputed for EFS and OS, respectively. Meta-analyses incorporating these studies did not significantly change the results (for EFS: HR = 1.83, 95% CI 1.54–2.19; for OS: HR = 1.87, 95% CI 1.45–2.40), which suggested the stability of the results (Fig 3). Sensitivity analyses revealed that no single study significantly altered the results (Fig 4).

## Discussion

In this meta-analysis, we demonstrated that positive MRD was associated with worse clinical outcomes in Ph+ ALL patients. The effect was consistent and robust in all subgroups that we were able to address, including studies that recruited patients of different age groups or disease status, used different therapies, cutoff levels and time points. In some subgroups, there were some preliminary indications that the effect of MRD status on EFS might be stronger than in others. These results should be interpreted with caution, since no significant differential subgroup effects were seen, and most of the confidence intervals overlapped. For OS, the prognostic value of MRD seemed to be greater in East Asian and USA patients than in European patients, and also greater in non-transplant cohorts than in transplant cohorts.

Recently there have been two meta-analyses that comprehensively analyzed the association of MRD and clinical outcomes in ALL patients [41, 42], which regarded Ph+ ALL patients as a subgroup in their analyses. Although their results uniformly indicated that MRD positivity was a negative marker in the Ph+ ALL subgroup, the number of studies included was very limited, which might influence the power of the results. In the meta-analysis of Berry et al [41], only five studies enrolling Ph+ ALL patients were included, with one study also included in our analysis. The other four studies were excluded because of including patients not treated with TKIs or less than 50 patients. The analysis of Bassan et al [42] included eleven studies in the Ph+ ALL subgroup. However, only four studies were also included in our analysis. The rest were excluded because of congress abstract, less than 50 patients or including patients not treated with TKIs. With our broad search strategy, we were able to include studies that investigated the prognostic value of MRD as many as possible, and restricted studies to TKIs treated cohorts. These ensured the reliability of the results, and allowed sufficient studies to be included for more detailed subgroup analyses.

We divided the timing of MRD measurement into four categories: before three months from induction, after three months from induction, pre-HSCT and post-HSCT. Studies that measured MRD before three months from induction mainly conducted the procedure at the end of induction or during early phase consolidation therapy. These fall into the first scenario raised by Bassan et al [42] that MRD measurement was to evaluate the quality of treatment response. Almost all studies that investigated the prognostic value of MRD taken after three months from induction conducted the measurement within six months from induction. These along with post-HSCT fall into the second scenario raised by Bassan et al [42] that MRD monitoring was used as a predictor for pending relapse. MRD negativity in the four categories all endowed benefit on EFS and OS. Although a larger effect was seen in the subgroup of after three months from induction, their confidence intervals overlapped.

With increasingly diverse approaches available for treating Ph+ ALL, there is particular interest in considering for whom allo-HSCT could be avoided. In a study of investigating the effects of nilotinib plus multi-agent chemotherapy in newly diagnosed Ph+ ALL [11], patients achieving complete molecular response (CMR) showed similar RFS between non-recipients and recipients of allo-HSCT. In another study by Wang et al [29], although there was benefit for allo-HSCT in the whole cohort, the effect was no longer evident for patients with low presenting white blood cell counts and 3-log reduction of BCR-ABL1 levels from baseline after two consolidation cycles. In a pediatric analysis [28], no significant difference existed between the transplant and non-transplant arms for OS and EFS in the standard-risk group defined as achieving CR at the induction end and major molecular response (MMR) at three months. These preliminary evidences suggested that MRD might play a role in sparing some low-risk Ph+ ALL patients for whom the toxic procedure of allo-HSCT might be dispensable.

Quantification of BCR-ABL1 transcript by RT-qPCR was used as the method to detect MRD by almost all the studies included in our meta-analysis. Various cutoff levels were used in different studies. The adverse impact of positive MRD was consistent in all cutoff level subgroups, although it should be noted that for the subgroup of ≥ 4-log reduction from baseline there was only one study, which contributed to non-significant confidence interval. The RT-qPCR assay is a convenient and straightforward approach for the detection of MRD in Ph+ ALL. However, BCR-ABL1 quantification in Ph+ ALL has yet been standardized like that in chronic myeloid leukemia (CML). Different approaches are used by individual physicians and national study groups. This contributed to the diverse rates and degrees of molecular response in clinical trials. Recently, the EURO-MRD consortium proposed guidelines for the work-flow and reporting of e1a2 BCR-ABL1 transcript levels in Ph+ ALL [43]. Their detailed laboratory recommendations provided a robust framework for the precise and reproducible RT-qPCR based analysis of e1a2 BCR-ABL1 transcript and will facilitate valid comparison of MRD results between clinical trials for Ph+ ALL.

Several limitations of the present meta-analysis should be mentioned. Firstly, emerging immunotherapy approaches such as CAR-T, blinatumomab and inotuzumab ozogamicin, and the potent third-generation TKI ponatinib have shown impressive efficacy in relapse/refractory or de novo Ph+ ALL. However, due to the limited data available, we were unable to address the role of MRD in these regimens treated cohorts. Secondly, some of the HRs were extracted from Kaplan-Meier curves, which might be less reliable than the original data or multivariate analysis results.

In conclusion, this meta-analysis indicated that MRD was a promising prognostic marker in the management of patients with Ph+ ALL. Overall, and in all subgroups analyzed, MRD positivity was associated with higher risk of relapse and mortality. The adverse impact appeared to be unaffected by variation in the timing or cutoff level of the MRD assay applied. Prospective trials using MRD-based risk stratification for patients with Ph+ ALL might elucidate the optimal post-remission management of these patients.

## Supporting information

S1 Checklist PRISMA 2009 checklist. (DOC)

S1 File Search strategy for Pubmed. (DOCX)

S2 File Raw data of this meta-analysis. (XLSX)

S1 Table Characteristics of the studies included in this meta-analysis. (DOCX)

S2 Table Quality assessment of the included studies using the QUIPS tool. (DOCX)

S3 Table Subgroup analyses of the impact of MRD status on the prognoses of Ph+ ALL. (DOCX)
