# Supplementary material for: Association of minimal residual disease with clinical outcomes in Philadelphia chromosome positive acute lymphoblastic leukemia in the tyrosine kinase inhibitor era: A systemic literature review and meta-analysis
Source: PLoS One. 2021 Aug 26;16(8):e0256801. doi: 10.1371/journal.pone.0256801 (PMC8389458; doi:10.1371/journal.pone.0256801)
Supplement: S1 Table — (DOCX) [file pone.0256801.s004.docx]

**S1 Table. Characteristics of the studies included in this meta-analysis.**

| **First author** | **Year** | **Region** | **Time** | **Study design** | **Treatment protocol** | **TKI** | **Number (with MRD)** | **Age (years)** |
| --- | --- | --- | --- | --- | --- | --- | --- | --- |
| Ottmann OG [17] | 2007 | Europe | 2002.05-2005.06 | RCT | chemo, TKI | imatinib | 55 (49) | 68 (54-79) |
| Yanada M [18] | 2008 | East Asia | NA | Prospective cohort | chemo, TKI, HSCT | imatinib | 100 (84) | 45 (15-64) |
| Lee S [19] | 2009 | East Asia | 2000.09-2006.02 | prospective cohort | chemo, TKI, HSCT | imatinib | 52 (52) | 35 (18-59) |
| Chen H [20] | 2012 | East Asia | 2005.05-2010.03 | prospective cohort | chemo, TKI, HSCT | imatinib | 82 (82) | 28 (3-51) |
| Ravandi F [21] | 2013 | USA | 2001.04-2011.03 | retrospective cohort | chemo, TKI | imatinib/dasatinib | 76 (55) | 54 (21-84) |
| Wang J [22] | 2014 | East Asia | 2006.05-2012.07 | retrospective cohort | chemo, TKI, HSCT | imatinib | 82 (76) | 38 (14-71) |
| Kim DY [11] | 2015 | East Asia | 2009.01-2012.05 | prospective cohort | chemo, TKI, HSCT | nilotinib | 90 (63) | 47 (17-71) |
| Short NJ [24] | 2016 | USA | 2001.04-2015.12 | retrospective cohort | chemo, TKI | imatinib/dasatinib/ponatinib | 85 (85) | 55 (21-80) |
| Rousselot P [9] | 2016 | Europe | 2007.08-2010.07 | prospective cohort | chemo, TKI, HSCT | dasatinib | 71 (49) | 69 (59-83) |
| Kuang P [26] | 2016 | East Asia | 2008.10-2012.06 | prospective cohort | chemo, TKI, HSCT | imatinib | 51 (35) | 36 (18-69) |
| Lussana F [23] | 2016 | Europe | NA | prospective cohort | chemo, TKI, HSCT | imatinib | 65 (65) | 43 (18-62) |
| Nishiwaki S [25] | 2016 | East Asia | 2002.09-2010.12 | retrospective cohort | chemo, TKI, HSCT | imatinib | 432 (432) | 43 (16-68) |
| Lou Y [7] | 2017 | East Asia | 2009.05-2015.12 | retrospective cohort | chemo, TKI, HSCT | imatinib | 153 (87) | 40 (18-68) |
| Bao XB [27] | 2017 | East Asia | 2006.07-2014.11 | retrospective cohort | chemo, TKI, HSCT | imatinib | 107 (98) | 30 (7-54) |
| Xue YJ [28] | 2018 | East Asia | 2010.08-2016.12 | retrospective cohort | chemo, TKI, HSCT | imatinib | 70 (68) | 10 (1-17) |
| Zhao X [30] | 2018 | East Asia | 2011.03-2016.06 | retrospective cohort | chemo, TKI, HSCT | imatinib | 155 (155) | 31 (4-63) |
| Wang J [29] | 2018 | East Asia | 2008.01-2016.07 | retrospective cohort | chemo, TKI, HSCT | imatinib | 145 (133) | 37 (14-65) |
| Pfeifer H [31] | 2018 | Europe | 2001.10-2009.11 | retrospective cohort | chemo, TKI, HSCT | imatinib | 97 (78) | 41 (18-64) |
| Yang F [32] | 2018 | East Asia | 2014.01-2017.06 | retrospective cohort | chemo, TKI, HSCT, CAR-T | imatinib/dasatinib | 63 (63) | 29 (15-61) |
| Fedullo AL [37] | 2019 | Europe | NA | retrospective cohort | chemo, TKI, HSCT | imatinib/dasatinib | 116 (116) | 51 (19-89) |
| Abou Dalle I [36] | 2019 | USA | 2001.04-2018.05 | retrospective cohort | chemo, TKI, HSCT | imatinib/dasatinib/ponatinib | 57 (41) | 50 (23-85) |
| Huang AJ [33] | 2019 | East Asia | 2007.01-2017.12 | retrospective cohort | chemo, TKI, HSCT | imatinib | 134 (134) | 38 (14-60) |
| Candoni A [34] | 2019 | Europe | 2005-2016 | retrospective cohort | chemo, TKI, HSCT | imatinib/dasatinib/nilotinib/ponatinib | 441 (404) | 43 (16-68) |
| Tang SH [35] | 2019 | East Asia | 2014.01-2017.01 | retrospective cohort | chemo, TKI, HSCT | imatinib/dasatinib | 63 (63) | NA |
| Li SQ [40] | 2020 | East Asia | 2011.03-2016.12 | retrospective cohort | chemo, TKI, HSCT | imatinib | 202 (202) | 32 (4-63) |
| Shen S [38] | 2020 | East Asia | 2015.01-2018.09 | RCT | chemo, TKI | imatinib/dasatinib | 189 (183) | 7.8 (IQR, 5.2-11.3) |
| Gu B [39] | 2020 | East Asia | 2016.01-2019.12 | retrospective cohort | CAR-T, HSCT | NA | 56 (51) | 34 (18-59) |

**S1 Table. Continued.**

| **First author** | **Ethnicity** | **Male/female** | **Follow-up** | **Sample** | **MRD timing** | **Disease status at MRD** | **Pre-MRD TX** | **Post-MRD TX** | **MRD test location** |
| --- | --- | --- | --- | --- | --- | --- | --- | --- | --- |
| Ottmann OG [17] | Caucasian | 25/30 | 11.2m (NA) | BM | NA | CR1 | chemo, TKI | chemo, TKI | central |
| Yanada M [18] | Asian | 55/45 | 3.2yrs (NA-5.1yrs) | BM | ≤ 3 months from induction | CR1 | chemo, TKI | chemo, TKI, HSCT | central |
| Lee S [19] | Asian | 29/23 | 49m (18-80m) | BM | ≤ 3 months from induction | CR1 | chemo, TKI | chemo, TKI, HSCT | central |
| Chen H [20] | Asian | 55/27 | 24.5m (4-72m) | BM | pre-HSCT | ≥ CR1 | chemo, TKI | HSCT | central |
| Ravandi F [21] | Caucasian | NA | 382w (232-501w) | BM | > 3 months from induction | CR1 | chemo, TKI | chemo, TKI | central |
| Wang J [22] | Asian | 50/32 | 25m (4-79m) | BM | ≤ 3 months from induction | CR1 | chemo, TKI | chemo, TKI, HSCT | central |
| Kim DY [11] | Asian | 45/45 | NA | PB | > 3 months from induction | CR1 | chemo, TKI | chemo, TKI, HSCT | central |
| Short NJ [24] | Caucasian | NA | 44m (5-171m) | NA | ≤ 3 months from induction | CR1 | chemo, TKI | chemo, TKI | central |
| Rousselot P [9] | Caucasian | 30/41 | 32m (2-88m) | BM, PB | > 3 months from induction | CR1 | chemo, TKI | chemo, TKI, HSCT | local |
| Kuang P [26] | Asian | 26/25 | 32m (4-74m) | BM | > 3 months from induction | CR1 | chemo, TKI | chemo, TKI, HSCT | local |
| Lussana F [23] | Caucasian | 31/34 | NA | BM | pre-HSCT | CR1 | chemo, TKI | HSCT | NA |
| Nishiwaki S [25] | Asian | 242/190 | 48m (2.2-125m) | NA | pre-HSCT | CR1 | chemo, TKI | HSCT | local |
| Lou Y [7] | Asian | 81/72 | 24.2m (0.5-76.3m) | BM | ≤ 3 months from induction | CR1 | chemo, TKI | chemo, TKI, HSCT | NA |
| Bao XB [27] | Asian | 64/43 | 19.8m (3.6-83.7m) | NA | pre-HSCT | ≥ CR1 | chemo, TKI | HSCT | central |
| Xue YJ [28] | Asian | 46/24 | 35.4m (1.2-90.2m) | BM | ≤ 3 months from induction | CR1 | chemo, TKI | chemo, TKI, HSCT | central |
| Zhao X [30] | Asian | 89/66 | NA | BM | post-HSCT | CR1 | HSCT | TKI | central |
| Wang J [29] | Asian | 80/65 | 33m (4-114m) | BM | ≤ 3 months from induction | CR1 | chemo, TKI | chemo, TKI, HSCT | central |
| Pfeifer H [31] | Caucasian | 58/39 | 35m (5.4-114.4m) | BM, PB | pre-HSCT | CR1 | chemo, TKI | HSCT | NA |
| Yang F [32] | Asian | 35/28 | 21m (5-47m) | BM | pre-HSCT | CR1 | chemo, TKI | HSCT | central |
| Fedullo AL [37] | Caucasian | 55/61 | NA | NA | NA | CR1 | chemo, TKI | chemo, TKI, HSCT | local |
| Abou Dalle I [36] | Caucasian | NA | 30m (12-125m) | NA | ≤ 3 months from induction | CR2 | chemo, TKI | chemo, TKI, HSCT | central |
| Huang AJ [33] | Asian | 71/63 | 32m (4-118m) | NA | ≤ 3 months from induction | CR1 | chemo, TKI | chemo, TKI, HSCT | local |
| Candoni A [34] | Caucasian | 230/211 | 39.4m (1-145m) | NA | pre-HSCT | ≥ CR1 | chemo, TKI | HSCT | local |
| Tang SH [35] | Asian | 35/28 | NA | BM, PB | ≤ 3 months from induction | CR1 | chemo, TKI | chemo, TKI, HSCT | local |
| Li SQ [40] | Asian | 117/85 | 1001d (24-2575d) | BM | pre-HSCT | CR1 | chemo, TKI | HSCT | central |
| Shen S [38] | Asian | 136/53 | 26.4m (2.1-50.6m) | NA | ≤ 3 months from induction | CR1 | chemo, TKI | chemo, TKI | central |
| Gu B [39] | Asian | 22/34 | 22m (3-48m) | BM | NA | ≥ CR2 | CAR-T | HSCT | central |

| **First author** | **MRD method** | **Cutoff** | **MRD positive (%)** | **Endpoint** | **Multi/Univariate** | **HR (95% CI)** |
| --- | --- | --- | --- | --- | --- | --- |
| Ottmann OG [17] | RT-qPCR | 10^-5^ | 28 (57.1%) | DFS | survival curve | 2.15 (0.94-4.93) |
| Yanada M [18] | RT-qPCR | 10^-5^ | 43 (51.2%) | RFS | survival curve | 1.25 (0.69-2.28) |
| Lee S [19] | RT-qPCR | ≥ 3 log reduction | 16 (30.8%) | DFS | multivariate | 4.60 (1.50-14.60) |
|  |  |  |  | OS | multivariate | 4.80 (1.50-14.70) |
| Chen H [20] | RT-qPCR | NA | 50 (61.0%) | DFS | multivariate | 3.70 (1.30-10.50) |
| Ravandi F [21] | RT-qPCR | 10^-3^ | 8 (14.6%) | OS | survival curve | 9.95 (2.74-36.18) |
| Wang J [22] | RT-qPCR | ≥ 1 log reduction | 42 (55.3%) | DFS | survival curve | 2.88 (1.12-7.37) |
|  |  |  |  | OS | survival curve | 2.52 (1.16-5.19) |
| Kim DY [11] | RT-qPCR | 10^-5^ | 9 (14.3%) | RFS | multivariate | 7.50 (1.90-29.58) |
| Short NJ [24] | RT-qPCR | 10^-4^ | 34 (40.0%) | RFS | multivariate | 2.44 (1.22-4.76) |
|  |  |  |  | OS | multivariate | 2.38 (1.22-4.76) |
| Rousselot P [9] | RT-qPCR | 10^-3^ | 13 (26.5%) | RFS | univariate | 2.27 (1.10-4.55) |
|  |  |  |  | OS | univariate | 1.85 (0.88-3.85) |
| Kuang P [26] | RT-qPCR | NA | 20 (57.1%) | DFS | multivariate | 4.32 (1.10-15.10) |
|  |  |  |  | OS | multivariate | 4.54 (0.99-19.20) |
| Lussana F [23] | RT-qPCR | ≥ 4 log reduction | 41 (63.1%) | DFS | survival curve | 1.04 (0.46-2.38) |
|  |  |  |  | OS | survival curve | 1.06 (0.46-2.42) |
| Nishiwaki S [25] | RT-qPCR | 10^-5^ | 155 (35.6%) | LFS | multivariate | 1.69 (1.27-2.24) |
|  |  |  |  | OS | multivariate | 1.58 (1.16-2.15) |
| Lou Y [7] | RT-qPCR | 10^-3^ | 22 (25.3%) | EFS | multivariate | 3.06 (1.29-7.30) |
|  |  |  |  | OS | multivariate | 5.38 (2.07-13.89) |
| Bao XB [27] | RT-qPCR | NA | 49 (50.0%) | LFS | multivariate | 2.79 (1.21-6.40) |
|  |  |  |  | OS | univariate | 1.25 (0.66-2.39) |
| Xue YJ [28] | RT-qPCR | ≥ 3 log reduction | 24 (35.3%) | EFS | multivariate | 8.41 (2.18-32.50) |
|  |  |  |  | OS | multivariate | 4.26 (1.27-14.31) |
| Zhao X [30] | RT-qPCR/FCM | 10^-4^ | 14 (9.7%) | LFS | multivariate | 3.61 (1.61-8.11) |
|  |  |  |  | OS | multivariate | 2.55 (1.01-6.44) |
| Wang J [29] | RT-qPCR | ≥ 3 log reduction | 51 (38.3%) | DFS | multivariate | 3.10 (1.70-5.90) |
|  |  |  |  | OS | multivariate | 3.50 (1.90-8.70) |
| Pfeifer H [31] | RT-qPCR | 10^-4^ | 27 (34.6%) | DFS | survival curve | 1.27 (0.64-2.53) |
|  |  |  |  | OS | survival curve | 1.01 (0.47-2.17) |
| Yang F [32] | RT-qPCR | ≥ 3 log reduction | 15 (23.8%) | EFS | multivariate | 2.91 (1.05-8.06) |
|  |  |  |  | OS | univariate | 3.82 (1.02-14.29) |
| Fedullo AL [37] | RT-qPCR | NA | 99 (85.3%) | DFS | multivariate | 2.49 (1.03-5.99) |
| Abou Dalle I [36] | RT-qPCR | 10^-3^ | 21 (51.2%) | RFS | multivariate | 2.08 (1.02-4.35) |
|  |  |  |  | OS | multivariate | 2.56 (1.06-6.25) |
| Huang AJ [33] | RT-qPCR | NA | 65 (48.5%) | DFS | univariate | 2.09 (1.54-2.83) |
|  |  |  |  | OS | univariate | 2.12 (1.52-2.98) |
| Candoni A [34] | RT-qPCR | NA | 257 (63.6%) | PFS | multivariate | 1.67 (1.23-2.27) |
|  |  |  |  | OS | multivariate | 1.54 (1.10-2.17) |
| Tang SH [35] | RT-qPCR | NA | 22 (34.9%) | OS | multivariate | 18.51 (4.77-71.73) |
| Li SQ [40] | FCM | 10^-5^ | 54 (26.7%) | LFS | survival curve | 1.47 (0.77-2.83) |
|  |  |  |  | OS | survival curve | 1.77 (0.92-3.37) |
| Shen S [38] | RT-qPCR | 10^-4^ | 39 (21.3%) | EFS | multivariate | 2.07 (0.84-5.10) |
| Gu B [39] | RT-qPCR | 10^-5^ | 13 (25.5%) | LFS | multivariate | 4.35 (1.96-10.00) |
|  |  |  |  | OS | multivariate | 3.13 (1.41-7.14) |

**S1 Table. Continued.**

**S1 Table. Continued.**

Abbreviations: NA, not available; RCT, randomized controlled trial; chemo, chemotherapy; TKI, tyrosine kinase inhibitor; HSCT, hematopoietic stem cell transplantation; CAR-T, chimeric antigen receptor T cell therapy; MRD, minimal residual disease; IQR, interquartile range; d, days; w, weeks; m, months; yrs, years; BM, bone marrow; PB, peripheral blood; CR1, the first complete remission; CR2, the second complete remission; TX, treatment.
