# Supplementary material for: Association of minimal residual disease with clinical outcomes in Philadelphia chromosome positive acute lymphoblastic leukemia in the tyrosine kinase inhibitor era: A systemic literature review and meta-analysis
Source: PLoS One. 2021 Aug 26;16(8):e0256801. doi: 10.1371/journal.pone.0256801 (PMC8389458; doi:10.1371/journal.pone.0256801)
Supplement: S2 Table — (DOCX) [file pone.0256801.s005.docx]

**S2 Table. Quality assessment of the included studies using the QUIPS tool.**

| **First author** | **Year** | **Study Participation** | **Study Attrition** | **Prognostic Factor Measurement** | **Outcome Measurement** | **Study Confounding** | **Statistical Analysis and Reporting** | **Total** |
| --- | --- | --- | --- | --- | --- | --- | --- | --- |
| Ottmann OG | 2007 | low | low | low | low | moderate | moderate | moderate |
| Yanada M | 2008 | low | low | low | low | moderate | moderate | moderate |
| Lee S | 2009 | low | low | low | low | low | low | low |
| Chen H | 2012 | low | low | low | low | low | low | low |
| Ravandi F | 2013 | low | low | low | low | moderate | moderate | moderate |
| Wang J | 2014 | low | low | low | low | moderate | moderate | moderate |
| Kim DY | 2015 | low | low | low | low | low | low | low |
| Short NJ | 2016 | low | low | low | low | low | low | low |
| Rousselot P | 2016 | low | moderate | low | low | moderate | moderate | moderate |
| Kuang P | 2016 | low | moderate | low | low | low | low | moderate |
| Lussana F | 2016 | low | low | low | low | moderate | moderate | moderate |
| Nishiwaki S | 2016 | low | low | low | low | low | low | low |
| Lou Y | 2017 | low | moderate | low | low | low | low | moderate |
| Bao XB | 2017 | low | low | low | low | low | low | low |
| Xue YJ | 2018 | low | low | low | low | low | low | low |
| Zhao X | 2018 | low | low | low | low | low | low | low |
| Wang J | 2018 | low | low | low | low | low | low | low |
| Pfeifer H | 2018 | low | low | low | low | moderate | moderate | moderate |
| Yang F | 2018 | low | low | low | low | low | low | low |
| Fedullo AL | 2019 | low | low | low | low | low | low | low |
| Abou Dalle I | 2019 | low | moderate | low | low | low | low | moderate |
| Huang AJ | 2019 | low | low | low | low | moderate | moderate | moderate |
| Candoni A | 2019 | low | low | low | low | low | low | low |
| Tang SH | 2019 | low | low | low | low | low | low | low |
| Li SQ | 2020 | low | low | low | low | moderate | moderate | moderate |
| Shen S | 2020 | low | low | low | low | low | low | low |
| Gu B | 2020 | low | low | low | low | low | low | low |
