# Supplementary material for: Association of minimal residual disease with clinical outcomes in Philadelphia chromosome positive acute lymphoblastic leukemia in the tyrosine kinase inhibitor era: A systemic literature review and meta-analysis
Source: PLoS One. 2021 Aug 26;16(8):e0256801. doi: 10.1371/journal.pone.0256801 (PMC8389458; doi:10.1371/journal.pone.0256801)
Supplement: S3 Table — (DOCX) [file pone.0256801.s006.docx]

**S3 Table.** **Subgroup analyses of the impact of MRD status on the prognoses of Ph+ ALL.**

|  | **EFS** | | | | | **OS** | | | | |
| --- | --- | --- | --- | --- | --- | --- | --- | --- | --- | --- |
|  | No. | metaHR (95% CI) | P_h_ | I^2^ (%) | Effect model | No. | metaHR (95% CI) | P_h_ | I^2^ (%) | Effect model |
| **Overall** | 25 | 2.00 (1.77-2.26) | 0.116 | 26.0 | F | 21 | 2.34 (1.86-2.95) | 0.002 | 53.4 | R |
| **Study design** |  |  |  |  |  |  |  |  |  |  |
| prospective | 9 | 2.08 (1.56-2.79) | 0.106 | 39.3 | F | 4 | 1.99 (1.24-3.19) | 0.126 | 47.6 | F |
| retrospective | 16 | 1.99 (1.74-2.27) | 0.205 | 21.8 | F | 17 | 2.38 (1.85-3.07) | 0.002 | 57.0 | R |
| **Age group** |  |  |  |  |  |  |  |  |  |  |
| 0-14 | 2 | 3.80 (0.97-14.82) | 0.091 | 65.1 | R | 1 | 4.26 (1.27-14.30) | / | / | / |
| 15-39 | 11 | 2.35 (1.93-2.86) | 0.346 | 10.3 | F | 10 | 2.28 (1.84-2.83) | 0.428 | 1.1 | F |
| ≥ 40 | 12 | 1.77 (1.51-2.08) | 0.354 | 9.4 | F | 9 | 1.94 (1.40-2.68) | 0.020 | 56.0 | R |
| **Region** |  |  |  |  |  |  |  |  |  |  |
| Europe | 6 | 1.69 (1.34-2.12) | 0.587 | 0.0 | F | 4 | 1.44 (1.10-1.89) | 0.584 | 0.0 | F |
| East Asia | 17 | 2.37 (1.91-2.96) | 0.060 | 37.5 | R | 14 | 2.68 (2.00-3.59) | 0.010 | 53.0 | R |
| USA | 2 | 2.26 (1.38-3.72) | 0.753 | 0.0 | F | 3 | 3.01 (1.83-4.96) | 0.143 | 48.5 | F |
| **Ethnicity** |  |  |  |  |  |  |  |  |  |  |
| Caucasian | 8 | 1.78 (1.44-2.19) | 0.666 | 0.0 | F | 7 | 1.84 (1.25-2.72) | 0.054 | 51.5 | R |
| Asian | 17 | 2.37 (1.91-2.96) | 0.060 | 37.5 | R | 14 | 2.68 (2.00-3.59) | 0.010 | 53.0 | R |
| **TKI** |  |  |  |  |  |  |  |  |  |  |
| 1st generation | 16 | 2.03 (1.65-2.50) | 0.091 | 33.9 | R | 13 | 2.08 (1.60-2.70) | 0.044 | 44.2 | R |
| 2nd generation | 2 | 3.55 (1.14-11.03) | 0.130 | 56.5 | R | 1 | 1.85 (0.88-3.87) | / | / | / |
| mix | 6 | 1.92 (1.52-2.43) | 0.803 | 0.0 | F | 6 | 3.62 (1.83-7.19) | 0.001 | 74.7 | R |
| NA | 1 | 4.35 (1.93-9.83) | / | / | / | 1 | 3.13 (1.39-7.04) | / | / | / |
| **Cohort** |  |  |  |  |  |  |  |  |  |  |
| transplant | 10 | 1.72 (1.45-2.03) | 0.354 | 9.6 | F | 9 | 1.58 (1.32-1.90) | 0.323 | 13.4 | F |
| non-transplant | 4 | 2.42 (1.57-3.71) | 0.814 | 0.0 | F | 3 | 3.41 (1.95-5.95) | 0.145 | 48.2 | F |
| mix | 11 | 2.37 (1.95-2.89) | 0.146 | 31.7 | F | 9 | 3.05 (2.17-4.30) | 0.084 | 42.5 | R |
| NA | 3 | 2.89 (1.78-4.69) | 0.457 | 0.0 | F | 1 | 3.13 (1.39-7.04) | / | / | / |

**S3 Table. Continued.**

|  | **EFS** | | | | | **OS** | | | | |
| --- | --- | --- | --- | --- | --- | --- | --- | --- | --- | --- |
|  | No. | metaHR (95% CI) | P_h_ | I^2^ (%) | Effect model | No. | metaHR (95% CI) | P_h_ | I^2^ (%) | Effect model |
| **MRD timing** |  |  |  |  |  |  |  |  |  |  |
| ≤ 3 months from induction | 10 | 2.27 (1.86-2.77) | 0.275 | 18.2 | F | 9 | 3.24 (2.29-4.60) | 0.085 | 42.3 | R |
| > 3 months from induction | 3 | 3.14 (1.78-5.55) | 0.276 | 22.4 | F | 3 | 3.91 (1.34-11.46) | 0.072 | 62.0 | R |
| pre-HSCT | 8 | 1.70 (1.43-2.03) | 0.447 | 0.0 | F | 7 | 1.50 (1.24-1.82) | 0.634 | 0.0 | F |
| post-HSCT | 1 | 1.48 (0.81-2.72) | / | / | / | 1 | 2.55 (1.01-6.44) | / | / | / |
| NA | 3 | 2.89 (1.78-4.69) | 0.457 | 0.0 | F | 1 | 3.13 (1.39-7.04) | / | / | / |
| **Disease status at MRD** | |  |  |  |  |  |  |  |  |  |
| CR1 | 20 | 1.99 (1.73-2.29) | 0.140 | 26.0 | F | 17 | 2.56 (1.93-3.39) | 0.003 | 56.0 | R |
| ≥ CR1 | 3 | 1.87 (1.42-2.47) | 0.218 | 34.4 | F | 2 | 1.47 (1.09-1.99) | 0.574 | 0.0 | F |
| CR2 | 1 | 2.08 (1.01-4.30) | / | / | / | 1 | 2.56 (1.05-6.22) | / | / | / |
| ≥ CR2 | 1 | 4.35 (1.93-9.83) | / | / | / | 1 | 3.13 (1.39-7.04) | / | / | / |
| **Cutoff** |  |  |  |  |  |  |  |  |  |  |
| 10^-5^ | 6 | 2.02 (1.37-3.00) | 0.055 | 53.8 | R | 3 | 1.73 (1.33-2.25) | 0.303 | 16.3 | F |
| 10^-4^ | 4 | 1.71 (1.20-2.42) | 0.540 | 0.0 | F | 3 | 1.81 (1.16-2.82) | 0.184 | 40.9 | F |
| 10^-3^ | 3 | 2.37 (1.53-3.68) | 0.790 | 0.0 | F | 4 | 3.55 (1.78-7.08) | 0.091 | 53.7 | R |
| ≥ 4 log reduction | 1 | 1.04 (0.46-2.37) | / | / | / | 1 | 1.06 (0.46-2.43) | / | / | / |
| ≥ 3 log reduction | 4 | 3.65 (2.32-5.74) | 0.556 | 0.0 | F | 4 | 3.92 (2.34-6.57) | 0.973 | 0.0 | F |
| ≥ 1 log reduction | 1 | 2.88 (1.12-7.39) | / | / | / | 1 | 2.52 (1.19-5.33) | / | / | / |
| NA | 6 | 2.03 (1.66-2.47) | 0.427 | 0.0 | F | 5 | 2.32 (1.36-3.97) | 0.004 | 74.5 | R |
| **Uni/multivariate** |  |  |  |  |  |  |  |  |  |  |
| survival curve | 6 | 1.47 (1.10-1.98) | 0.563 | 0.0 | F | 5 | 1.92 (1.04-3.56) | 0.024 | 64.5 | R |
| univariate | 2 | 2.12 (1.60-2.80) | 0.834 | 0.0 | F | 4 | 1.94 (1.48-2.55) | 0.378 | 2.8 | F |
| multivariate | 17 | 2.44 (1.97-3.02) | 0.099 | 32.1 | R | 12 | 2.91 (2.06-4.09) | 0.004 | 60.2 | R |

Abbreviations: NA, not available; Ph, P value for heterogeneity; F, fixed-effects model; R, random-effects model.
